# Obturator inlet and iliac oblique fluoroscopic views allow for placement of longer iliac screws

**DOI:** 10.1016/j.xnsj.2025.100751

**Published:** 2025-06-13

**Authors:** Monty Khela, Obiajulu Agha, Mark Xu, Neel Anand, David Gendelberg, Ashraf N. El Naga

**Affiliations:** a School of Medicine, Creighton University, Omaha, NE, USA; b Department of Orthopaedic Surgery, University of California San Francisco (UCSF), San Francisco, CA, USA; c Department of Orthopaedic Surgery, Cedars-Sinai Medical Center, Los Angeles, CA, USA

**Keywords:** Iliac screw placement, Spinopelvic fixation, Spinal fusion surgery, Fluoroscopy-guided surgery, OIIO technique, Spinal instrumentation

## Abstract

**Background:**

Achieving strong distal fixation in posterior spinal fusion (PSF) surgeries sometimes necessitates iliac screw placement. However, traditional methods such as freehand and CT navigation techniques face limitations concerning screw length, accuracy, ergonomic challenges, surgical time, and drill deflection. Fluoroscopic iliac screw guidance using the obturator inlet and iliac oblique (OIIO) views may mitigate some of these drawbacks.

**Methods:**

This retrospective comparative study was conducted at a university-affiliated tertiary care hospital and Level 1 trauma center, reviewing cases from January 2020 to December 2022. A total of 119 patients who underwent posterior spinal fusion with pelvic fixation were included and categorized into 3 groups: OIIO (*n* = 20), freehand (*n* = 46), and CT navigation-assisted (*n* = 53). Screw length, diameter, and cortical breach rates were assessed using intraoperative and postoperative imaging. Statistical analyses were performed using 1-way ANOVA and Fisher’s Exact test (p < .05).

**Results:**

Screw lengths were 97.95 ± 6.95 mm, 78.68 ± 5.17 mm, and 85.27 ± 7.01 mm in the OIIO, freehand, and navigation groups, respectively (p < .0001). Despite the longer screws, the OIIO group had no cortical breaches, compared to 0.58% in the freehand group and 0% in the CT navigation group (p = .5683). No significant differences were observed in iliac screw revision rates across groups (p = .6175), suggesting the OIIO technique is not inferior to freehand and navigation-assisted methods regarding safety and screw length.

**Conclusions:**

The OIIO technique allows for favorable screw sizes compared to freehand and navigation-assisted methods without increasing cortical breach risk. This technique provides a safe and ergonomic alternative, particularly in settings where advanced navigation technology is unavailable.

## Introduction

Posterior spinal instrumentation often requires extension to the pelvis to achieve robust distal fixation, especially in complex trauma, deformity, and degenerative conditions [1,2]. The iliac screw is a common tool used to secure this fixation, taking advantage of the robust bony corridor spanning from the anterior inferior iliac spine (AIIS) to the posterior superior iliac spine (PSIS), commonly referred to as the supraacetabular corridor or sciatic buttress. Accurate placement of these screws is crucial to avoid complications such as neurovascular injury or joint penetration [3].

Traditionally, iliac screws have been placed using freehand techniques confirmed by fluoroscopic views such as the obturator outlet (teardrop view) or via CT navigation. The freehand technique relies heavily on anatomical landmarks and palpation, which can be imprecise due to variations in patient anatomy and habitus, leading to potential breaches of the iliac cortices or the use of shorter screws in order to avoid a breach. While CT navigation offers enhanced accuracy by providing detailed 3D guidance, it is not always available, increases radiation exposure, and can prolong surgical time [4,5]. Further, navigation relies on a projection of the drill and may not account for deflection of a drill off the sacroiliac joint [6].

**Fig. 1:**
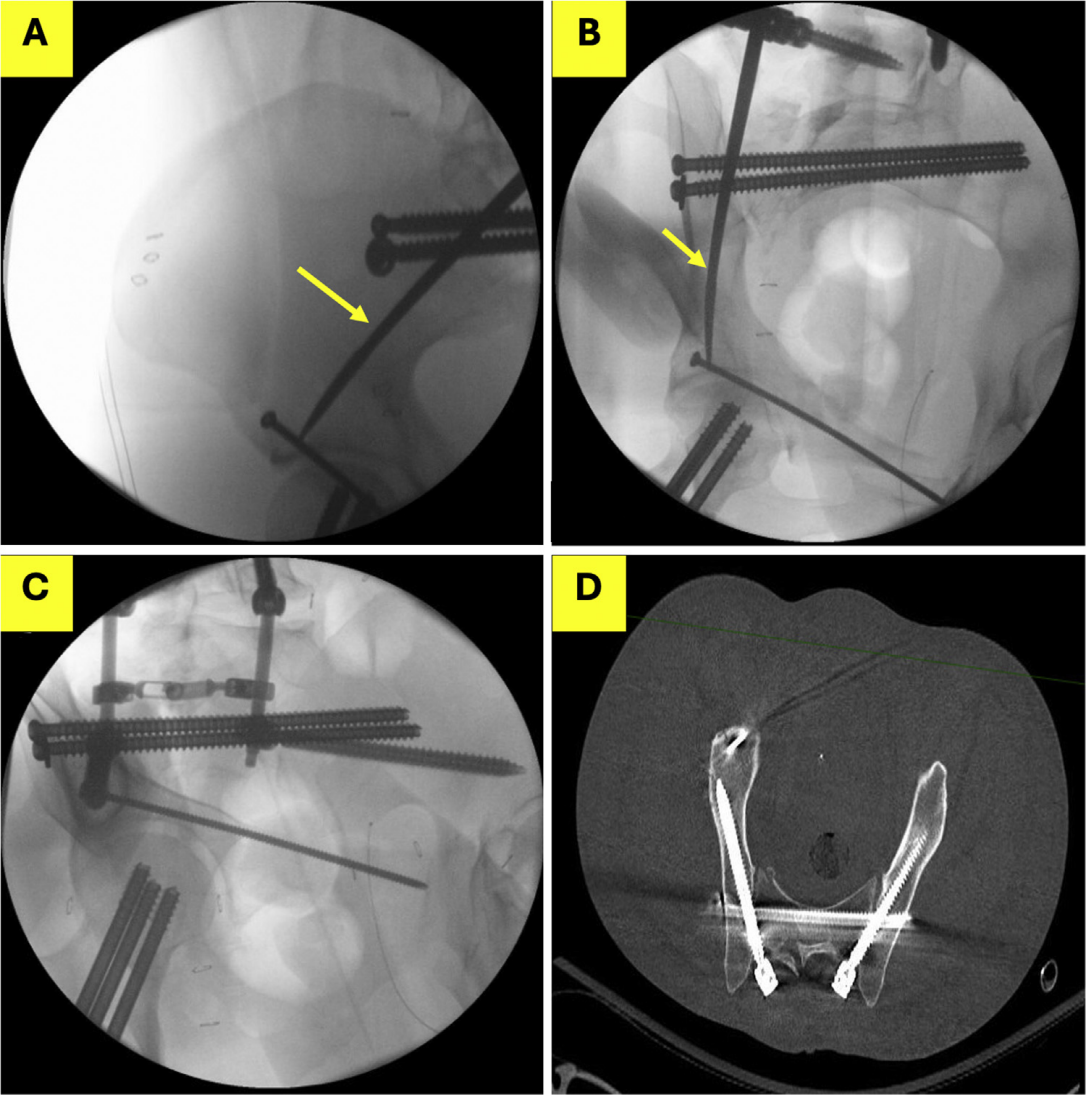
A 42-year-old patient undergoing percutaneous lumbopelvic fixation for a complex pelvic ring fracture. (A) Iliac oblique view guiding the cranial/caudal trajectory of the pedicle finder (yellow arrow). The pedicle finder is cranial to the sciatic notch and directed towards the distal aspect of the anterior inferior iliac spine. (B) Obturator inlet view guiding the medial-lateral trajectory of the pedicle finder. (C) The obturator outlet (“teardrop”) view is used as a confirmatory view after placement of the right iliac screw. (D) Postoperative CT demonstrates safe intraosseus placement of the pelvic screw occupying the length of the corridor.

Recognizing the limitations of these traditional approaches, our team has employed the obturator inlet and iliac oblique (OIIO) fluoroscopic technique as previously reported [6]. The OIIO method leverages the obturator inlet and iliac oblique views to guide screw placement, allowing for full visualization of the iliac corridor while optimizing the positioning of the fluoroscopy C-arm (Fig. 1) with the obturator inlet view guiding the medial-lateral trajectory, and the iliac oblique guiding the cranial caudal trajectory of the screw. This technique facilitates the placement of either S2AI of iliac screws without the ergonomic challenges posed by the teardrop view, where the fluoroscope often obstructs the surgeon's access to the surgical site [7].

The objective of this study is to compare the OIIO technique with the conventional freehand and navigated screw placement methods. Specifically, we aim to evaluate the length and diameter of the screws placed using these techniques and assess the incidence of cortical breaches. The study's significance lies in its potential to assess whether the ergonomic OIIO technique is a safe and reliable method to place long iliac screws, particularly in settings where advanced navigation technology is unavailable [7,8].

## Materials and methods

This retrospective comparative study was conducted at 2 academic hospitals affiliated with the same university, encompassing patients who underwent PSF with pelvic fixation between January 2020 and December 2022. The study was approved by the Institutional Review Board.

Patients were eligible for inclusion if they had undergone PSF with iliac screw placement using 1 of 3 techniques: the OIIO technique, traditional freehand technique, or CT navigation-assisted technique. Exclusion criteria included incomplete medical records or prior revision surgeries before the study period. A total of 119 patients met the inclusion criteria, with 20 in the OIIO group, 46 in the freehand group, and 53 in the CT navigation group.

The primary outcomes assessed were screw length, screw diameter, and the incidence of cortical breaches. Secondary outcomes included the rate of subsequent iliac screw revisions. Data was collected from patient medical records, intraoperative fluoroscopic images, and postoperative CT scans. Screw measurements were standardized across all groups to ensure comparability, and cortical breaches were identified by reviewing both intraoperative and postoperative imaging.

Efforts to minimize bias, secondary to the large discrepancy in sample size among the groups, included the random selection of patients for the non-OIIO groups and the use of standardized protocols for data collection and analysis. The study size of the non-OIIO group was determined based on the number of patients who met the inclusion criteria within the specified period, ensuring sufficient power to detect significant differences between groups.

Quantitative variables such as screw length and diameter were analyzed as continuous variables, and comparisons were made using 1-way ANOVA. Categorical variables, including the incidence of cortical breaches, were analyzed using Fisher’s Exact test. Statistical analyses were conducted in GraphPad Prism version 10 software, with significance set at p < .05.

## Results

### Patient demographics and surgical details

A total of 119 patients were included in this study. Twenty patients who underwent iliac screw placement using the OIIO technique were compared with 99 patients who received traditional screw placement, either through freehand (*n* = 46) or CT navigation-assisted methods (*n* = 53). The OIIO group consisted of 39 screws, while the non-OIIO group included 173 screws placed freehand and 113 screws placed with navigation assistance. Patients in the OIIO group were significantly younger than those in the freehand and navigation groups (mean age 53.75 ± 20.79 years vs. 77.05 ± 2.75 years vs. 78.00 ± 3.53 years, respectively; p < .0001).

### Screw length and diameter

**Fig. 2:**
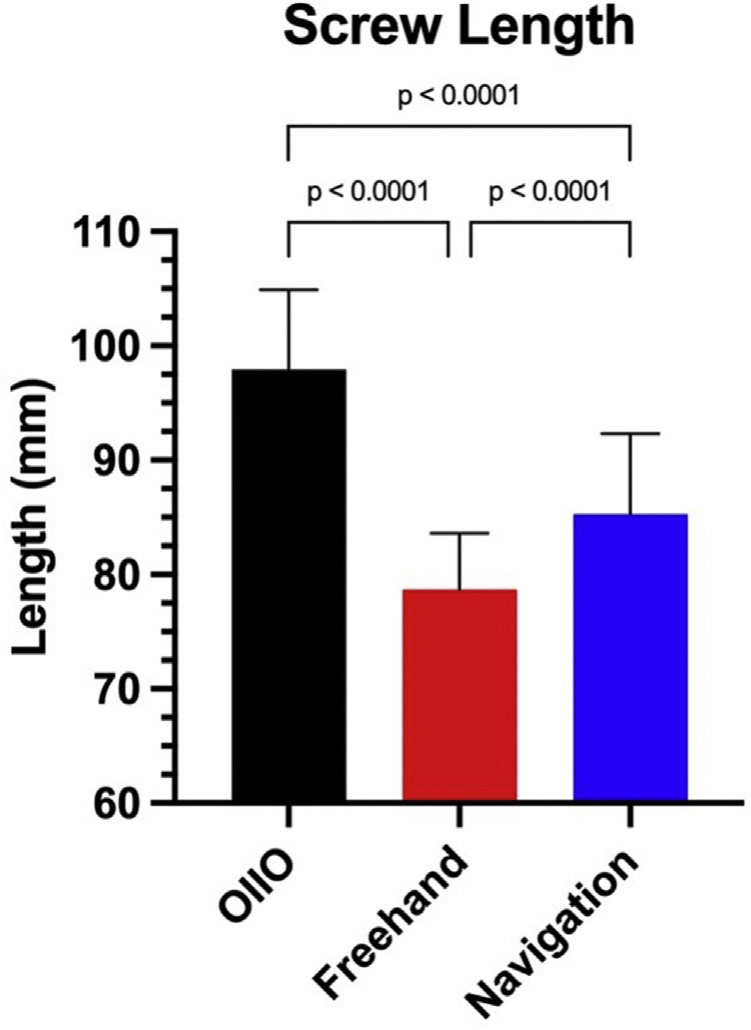
Comparison of screw lengths across different surgical techniques. The bar graph shows the mean screw lengths (mm) for the OIIO, freehand, navigation-assisted technique. The OIIO technique demonstrates significantly longer screws in comparison with freehand and navigation.

The screws placed using the OIIO technique were longer compared to those placed using the freehand and navigation techniques (Fig. 2). The mean screw length for the OIIO group was 97.95 ± 6.95 mm, compared to 78.68 ± 5.17 mm for freehand screws (p < .0001) and 85.27 ± 7.01 mm for navigated screws (p < .0001).

**Fig. 3:**
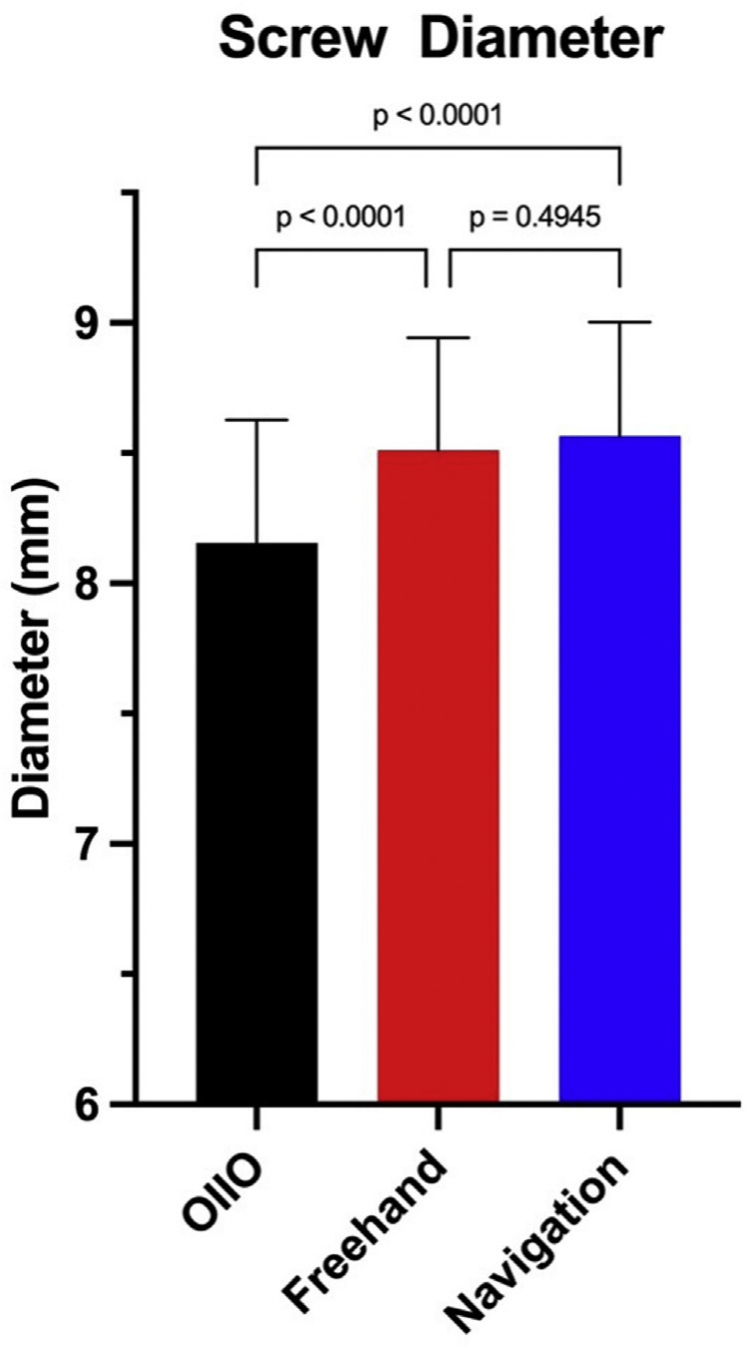
Comparison of screw diameters across different surgical techniques. The bar graph shows the mean screw diameters (mm) for the OIIO, freehand, and navigation-assisted technique. The freehand and navigation techniques demonstrated significantly larger screw diameters in comparison with OIIO.

In terms of screw diameter, OIIO screws were slightly narrower than those placed using both the freehand and navigation techniques (Fig. 3). The mean screw diameter in the OIIO group was 8.16 ± 0.47 mm, compared to 8.51 ± 0.42 mm for freehand screws (p < .0001) and 8.56 ± 0.41 mm for navigated screws (p < .0001).

### Cortical breaches and revisions

There were no instances of cortical breaches in the OIIO group. In contrast, one instance of lateral cortical breach occurred in the freehand group, where the screw violated the lateral iliac cortex (p = .5683). No cortical breaches were reported in the navigation group. Furthermore, there were no significant differences in the rates of subsequent iliac screw revision between the OIIO group and the non-OIIO group (p = .6175).

## Discussion

The findings from this study provide compelling evidence that the OIIO fluoroscopic technique is noninferior to traditional freehand and CT navigation-assisted techniques for iliac screw placement in posterior spinal fusion. The OIIO technique not only enables the placement of longer screws but also does so without an increased risk of cortical breaches, a critical factor in ensuring patient safety during and after surgery [[8], [9], [10]].

Traditional methods of iliac screw placement, such as the freehand technique, rely heavily on anatomical landmarks and fluoroscopic confirmation through the teardrop view (obturator outlet). While this method is widely used, it poses challenges, particularly in terms of screw length and the risk of cortical breaches. Palpation of external landmarks such as the greater trochanter is notably nonspecific in patients of varying body habitus. Additionally, when the cortex is reached and palpated with a probe, it sometimes remains unclear how to redirect the trajectory to allow for placement of a longer screw. When the teardrop view is used, it is often as a confirmatory view. With the C-arm in position to acquire the “teardrop” it often obstructs the surgeon’s access to the surgical site, especially with the length of the instrumentation tools, making it difficult to achieve optimal screw placement without compromising safety [[11], [12], [13]]. This limitation is particularly pronounced in cases where patient anatomy is atypical, when guidance is desired for directing trajectory, or where existing hardware further complicates the surgical field [14,15].

CT navigation, while more accurate and allowing for visualization of the entire available corridor, is not without its drawbacks. It requires advanced imaging technology that may not be available in all surgical settings, particularly in lower-resource areas. Additionally, the use of CT navigation increases radiation exposure to both the patient and can extend the duration of surgery [6]. The results of our study highlight that the OIIO technique offers a viable alternative by combining the benefits of enhanced visualization with reduced ergonomic challenges and no additional radiation exposure. Further, navigated techniques rely on projections of instruments and does not account for deflection of instruments such as a narrow diameter drill bit which can deflect off the hard cortical bone of the iliosacral joint for instance.

The ability to place longer screws using the OIIO technique as compared to the freehand technique is particularly noteworthy. Longer screws provide more robust fixation, which is essential in complex cases such as spinal deformity, trauma, and severe degenerative disease [8,10,11]. In their biomechanical study evaluating insertional torque, Santos et al. [16] demonstrated that the greatest mean insertional toques were seen at depths greater than 80 mm from the posterior superior iliac spine. This is particularly relevant in situations where achieving optimal biomechanical stability is crucial to the success of the surgical outcome. Our study found a small difference with regards to the narrower diameter of the screws placed using the OIIO technique. While statistically significant, this is unlikely to have a clinically significant impact on the strength of the fixation based on the available literature, as the difference is less than half a millimeter.

The absence of cortical breaches in the OIIO group is a significant finding, underscoring the safety of this technique. Cortical breaches can lead to serious complications, including damage to neurovascular structures, which can result in debilitating outcomes for patients [17]. The fact that no cortical breaches were observed in the OIIO group, despite the placement of longer screws, suggests that this technique may reduce the risk of such complications compared to both freehand and navigation-assisted methods.

One of the most critical aspects of the OIIO technique is its accessibility. Unlike CT navigation, which requires expensive and sophisticated equipment, the OIIO technique relies on standard fluoroscopy, which is widely available in most surgical centers around the world. This makes the OIIO technique a particularly valuable option in resource-limited settings, where access to advanced imaging technology is not feasible. By offering a method that does not compromise on safety or effectiveness, the OIIO technique can play a crucial role in improving surgical outcomes in diverse healthcare environments.

Furthermore, the ergonomic benefits of the OIIO technique cannot be overstated. The ability to position the C-arm in a way that does not obstruct the surgeon's access to the surgical field reduces the physical strain on the surgical team and allows for more precise and controlled movements. This is particularly important in lengthy surgeries, where surgeon fatigue can lead to errors.

While the results of this study are promising, there are some limitations that should be acknowledged. The retrospective nature of the study introduces the possibility of selection bias, and the relatively small sample size, particularly in the OIIO group, may limit the generalizability of the findings. Selection bias is of particular concern as the driving difference between the patients undergoing the different instrumentation techniques. As the technique used was determined by the treating surgeon, the differences in screw lengths may be related to differences in the goals of the surgeons using the different techniques. Nevertheless, use of the OIIO technique allowed for placement of screws at least comparable to the other techniques. Future studies with larger sample sizes and prospective designs are needed to confirm the safety and efficacy of the OIIO technique across a broader patient population.

In addition, while the OIIO technique appears to be a safe and effective alternative to traditional methods, further research is needed to explore its reproducibility, long-term outcomes, particularly in terms of screw loosening, hardware failure, and patient-reported outcomes. Studies comparing the OIIO technique with other emerging technologies, such as robotic-assisted screw placement, would also be valuable in determining its relative advantages in different clinical scenarios.

## Conclusion

The OIIO fluoroscopic technique for iliac screw placement allows for a reproducible method to guide the medial-lateral and cranial caudal trajectory of iliac fixation in order to allow for maximal utilization of this robust bony corridor. By enabling the placement of longer screws without an increased risk of cortical breaches, the OIIO technique has the potential to improve surgical outcomes, particularly in complex cases where robust fixation is essential. Its widespread applicability, even in resource-limited settings, further underscores its value as a versatile tool in the spine surgeon's arsenal.

## Funding

No financial support was provided for this study.

## Declaration of competing interests

One or more of the authors declare financial or professional relationships on ICMJE-NASSJ disclosure forms.
